# ALZHEIMER’S DISEASE RISK REDUCTION HEALTH COACHING: ALCOHOL EFFECTS

**DOI:** 10.1093/geroni/igac059.524

**Published:** 2022-12-20

**Authors:** Faika Zanjani, Annie Rhodes, Brian Battle

**Affiliations:** Virginia Commonwealth University, Richmond, Virginia, United States; Virginia Commonwealth University, Richmond, Virginia, United States; VCU, Richmond, Virginia, United States

## Abstract

Lifestyle risk reduction at the community-level, is currently considered an effective method to decrease Alzheimer’s disease (AD). As part of the Virginia Commonwealth University iCubed Health and Wellness in Aging Core, diverse older adults (60+) in Richmond, VA, with incomes below $12,000/year and managing either diabetes/cardiovascular symptoms, were offered weekly lifestyle telephone-health coaching for 12-weeks, providing education, support, and monitoring for AD lifestyle risk in 2020-21. The study sample (n=40, mean age 68 years (range: 60-77 years) was 88% African American/Black (n=35), 100% Non-Hispanic, 45% males (n=18)), 60% reporting memory problems and 53% reporting any alcohol consumption. Thirty-nine (95%) of subjects successfully participated in coaching sessions; on average 91.9% (11) sessions were completed. Participants provided positive anecdotal feedback and the need for continued coaching during COVID. Average drinks per day decreased across the study period (F=7.44; p=.01) and alcohol risk, defined as more than 1 drink/day, decreased (F=3.46;p=.07). Drinking at baseline was associated with differential change in nicotine dependence (F=14.00 ;p=.02), depression risk (F=3.20;p=.09), light physical activity (F=4.52;p=.05), and cognition (COGTEL) (F=6.35;p=.02). Drinking between-subject effects indicated poorer level differences for smoking risk (F=5.68;p=.02), physical inactivity risk (F=4.66;p=.04), and total health behavior risk (F=14.54;p=.001), but higher cognition-scores (F=3.18;p=.11) for drinkers. In conclusion, there may be a paradoxical health effect for alcohol, with associations for negative health behaviors, but positive cognitive functioning. In conclusion, this preliminary work creates the impetus for future large-scale AD risk reduction investigations to improve the lives of AD-risk, low-income, diverse older adults reporting alcohol consumption.

